# Chatbot to Support the Mental Health Needs of Pregnant and Postpartum Women (Moment for Parents): Design and Pilot Study

**DOI:** 10.2196/72469

**Published:** 2025-04-08

**Authors:** Kelsey McAlister, Lara Baez, Jennifer Huberty, Marianna Kerppola

**Affiliations:** 1 Fit Minded Inc, 14848 N 46th Place, Phoenix, AZ, 85032, United States, 1 (602) 935-6986; 2 Poisera Inc (dba Moment for Parents), Ann Arbor, MI, United States

**Keywords:** perinatal support, human-centered design, digital health, maternal health, chatbot, digital tool

## Abstract

**Background:**

Maternal mental health disorders are prevalent, yet many individuals do not receive adequate support due to stigma, financial constraints, and limited access to care. Digital interventions, particularly chatbots, have the potential to provide scalable, low-cost support, but few are tailored specifically to the needs of perinatal individuals.

**Objective:**

This study aimed to (1) design and develop Moment for Parents, a tailored chatbot for perinatal mental health education and support, and (2) assess usability through engagement, usage patterns, and user experience.

**Methods:**

This study used a human-centered design to develop Moment for Parents, a rules-based chatbot to support pregnant and postpartum individuals. In phase 1, ethnographic interviews (n=43) explored user needs to inform chatbot development. In phase 2, a total of 108 pregnant and postpartum individuals were recruited to participate in a pilot test and had unrestricted access to the chatbot. Engagement was tracked over 8 months to assess usage patterns and re-engagement rates. After 1 month, participants completed a usability, relevance, and satisfaction survey, providing key insights for refining the chatbot.

**Results:**

Key themes that came from the ethnographic interviews in phase 1 included the need for trusted resources, emotional support, and better mental health guidance. These insights informed chatbot content, including mood-based exercises and coping strategies. Re-engagement was high (69/108, 63.9%), meaning users who had stopped interacting for at least 1 week returned to the chatbot at least once. A large proportion (28/69, 40.6%) re-engaged 3 or more times. Overall, 28/30 (93.3%) found the chatbot relevant for them, though some noted repetitive content and limited response options.

**Conclusions:**

The Moment for Parents chatbot successfully engaged pregnant and postpartum individuals with higher-than-typical retention and re-engagement patterns. The findings underscore the importance of flexible, mood-based digital support tailored to perinatal needs. Future research should examine how intermittent chatbot use influences mental health outcomes and refine content delivery to enhance long-term engagement and effectiveness.

## Introduction

Maternal mental health disorders are a critical public health concern due to their high and rising prevalence. The World Health Organization (WHO) estimates that 10% of pregnant women and 13% of postpartum women develop a mental health disorder [1]. In the United States, 1 in 5 women experience a mental health disorder during pregnancy or within the first year after birth [2]. While postpartum depression is the most prevalent, affecting approximately 1 in 8 women [3], other maternal mental health disorders affect women, including anxiety, psychosis, obsessive-compulsive disorder, bipolar disorder, posttraumatic stress disorder, and schizophrenia [4]. These challenges are even more pronounced among underrepresented populations, who experience higher rates of maternal mood disorders [5,6]. Although treatable, 75% of women with maternal mental health disorders go untreated [2], which poses serious health concerns for both the mother and the infant. Untreated maternal mental health conditions are linked to increased risks for preterm birth, low birth weight, developmental delays, and impaired maternal-infant bonding, and also raise the mother’s risk of substance use, psychosis, and suicide [4,7]. The lack of ongoing education and support of mental health issues during and after pregnancy often leaves these women without timely support or interventions, exacerbating the problem.

There are several barriers to care contributing to untreated maternal mental health disorders, including stigma, lack of access to mental health care professionals, and high-cost challenges [8-11]. These challenges are especially prevalent in underrepresented populations, such as Black women, who often face additional obstacles such as systemic racism, cultural insensitivity, and mistrust of health care providers [12-14]. Other barriers include reluctance to acknowledge symptoms and a lack of support from partners, family members, and health care professionals [11,15]. Social pressures and stigma often prevent women from discussing their feelings openly, leading to feelings of shame and fear of losing custody of their child [15]. Additionally, many mothers have limited knowledge of maternal mental health problems, hindering their ability to recognize symptoms and seek timely help [10,11]. In some cases, individuals may turn to online sources or personal networks for support [16,17], but these resources can be incomplete or inaccurate. Without adequate support, these barriers perpetuate high rates of untreated maternal mental health disorders, negatively affecting both mother and child health outcomes.

Digital tools are a promising solution to addressing barriers to maternal mental health care. These tools offer several advantages, including scalability, affordability, and accessibility, and provide real-time support without the stigma associated with in-person care. A wealth of digital tools for perinatal mental health have been tested in clinical trials, and a recent meta-analysis found that these tools are modestly effective in reducing symptoms of anxiety and depression [18]. However, challenges have emerged in engaging and retaining users of many digital tools due to the excess burden they place on users (eg, needing to download and learn to use a new mobile app). Chatbots have emerged recently as a way to engage perinatal users with digital interventions using a mode of communication that is ubiquitous and comfortable (eg, texting) [19]. Furthermore, chatbots simulate conversations with users, which may help them stay engaged [20]. Chatbots are promising because they offer cost-effective, real-time engagement [21,22]. Chatbots monitor mental health symptoms longitudinally, with each conversation functioning as an ecological momentary assessment, capturing real-time insights into emotional well-being and enabling timely interventions [23]. Importantly, chatbots promote honest disclosure for emotional well-being [24,25].

The success of these tools depends on adopting a human-centered approach, which ensures that the tool aligns with the specific needs and preferences of its users. A human-centered design (HCD) approach involves feedback from users during development, ensuring that interactions are empathetic, personalized, and accessible [26]. HCD integrates human factors, ergonomics, and usability techniques to enhance product design [26,27]. Additionally, it often involves ethnographic research and strategies that explore how individuals interact with systems, ensuring products are safer, easier to use, and aligned with the intended users’ needs and abilities [26,27]. This approach helps create engaging experiences, fostering trust and consistent use, which are critical for maintaining long-term effectiveness in mental health support [26].

A growing body of research has demonstrated the utility of engaging in HCD products when developing tools for perinatal populations. Evidence shows that perinatal women widely use and value health-related apps, particularly for support, education, and self-monitoring. For example, many women find apps helpful for personalized health tracking, connecting with online communities, and addressing mental health concerns [28,29]. Furthermore, studies indicate that apps are most effective when designed with input from users and health care professionals, addressing gaps such as information reliability, security, and clinical validation [30,31]. HCD approaches have shown high feasibility and satisfaction, emphasizing the importance of tailoring interventions to the specific challenges and preferences of this population [32].

Despite the potential of digital tools, there remains a critical gap in solutions that offer both education and meaningful mental health support for pregnant and postpartum individuals. Many chatbots use evidence-based techniques such as cognitive behavioral therapy but are not designed for the unique needs of perinatal populations, often failing to address fluctuating emotions, stigma, and cultural sensitivities [33,34]. Additionally, these tools frequently lack longitudinal support and proactive interventions for perinatal mood and anxiety disorders [35]. Therefore, the purpose of this study was to develop and evaluate a chatbot called Moment for Parents. The goal of Moment for Parents is to address the current need for a digital mental health intervention to support the needs of pregnant and postpartum people. This study aimed to (1) design and develop a chatbot tailored specifically to educate and support pregnant and postpartum people on emotional well-being and mental health and (2) evaluate the usability of Moment for Parents in a pilot test by examining usage patterns, engagement levels, and user experience.

## Methods

### Design and Development

This study used a HCD approach to develop the Moment for Parents chatbot. HCD is an interactive system development approach that aims to create useful products by focusing on users, their needs, and their requirements [26,36]. HCD leads to inclusive health technology that improves mental health equity because the system engages users to improve adherence, adoption, and effectiveness [26,27,37]. By applying these principles, we aimed to develop a more inclusive and supportive chatbot for the mental health and well-being of pregnant and postpartum end users.

**Figure 1.**
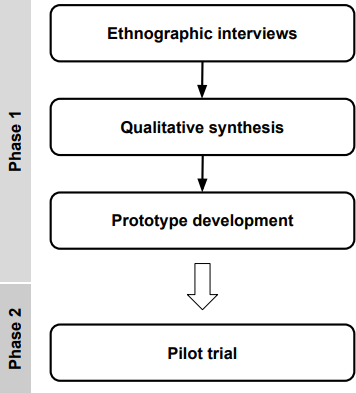
Overview of this study’s design.

Figure 1 depicts the Moment for Parents study design. In phase 1, we engaged in ethnographic interviews with pregnant and postpartum end users to understand their needs and preferences. These findings informed the initial prototype of the Moment for Parents chatbot. In phase 2, the initial prototype was launched for pilot-testing to evaluate its usability, functionality, and relevance to users’ needs. Throughout phase 2, the Moment for Parents chatbot experience was iteratively updated as participants provided feedback.

### Ethical Considerations

This study is a retrospective analysis of secondary data, and therefore, it was exempt from additional consent requirements under human subjects regulations and received approval from the BRANY (Biomedical Research Alliance of New York) Institutional Review Board (study ID 24-338-1949). All study data were deidentified. Participants did not receive compensation for participation.

### Phase 1 – Ethnographic Interviews

#### Participants

A total of 43 participants were recruited via snowball sampling, starting with existing networks and communities and expanding through participant referrals. Participants were eligible to participate in this study if they self-reported being pregnant or up to 1 year post partum.

#### Procedure

Data collection took place between March and November 2019. Ethnographic interviews were conducted via the videoconferencing service Zoom (Zoom Communications, Inc). The same interviewer conducted all interviews. Interviews were recorded with participant permission, and the interviewer took reflexive notes throughout the interviews.

#### Measures

Of ethnographic interview, participants were asked to provide their gestational age if pregnant or the age of their youngest child if post partum. Participants were also asked how many additional children they had and their ages. No other demographic information was collected from participants. The ethnographic interviews were designed to explore participants’ mental health needs, challenges during pregnancy or post partum, and expectations for digital support tools. Questions addressed a range of topics, including emotional well-being, resources they have found helpful, and desired features for a mental health chatbot. Sample questions included:

*How has your pregnancy (if pregnant) been going so far? What have been your go-to resources to stay sane during pregnancy? What tools and/or resources were the most helpful?? After you had your baby (if postpartum), what tools or resources were the most helpful postpartum? Is there anything else you wish you had to help prepare for birth/motherhood? As you reflect on your pregnancy and postpartum experience, how did you think about self-care? What were the biggest hurdles/challenges in taking care of yourself?*

In addition to ethnographic interviews, additional input was collected from a multidisciplinary team of experts, including yoga and meditation instructors, midwives, doulas, public health professionals, and therapists specializing in maternal mental health. Insights for the Moment for Parents chatbot were also drawn from relevant maternal mental health literature, literature reviews on chatbots, and the team’s lived experience.

#### Data Analysis

Audio recordings of interviews were transcribed, and reflexive interview notes were reviewed. Data were analyzed using a qualitative description approach [38], wherein the coder identified themes and subthemes relevant to the Moment for Parents prototype development.

### Phase 2 – Pilot Test

#### Participants

A new sample of participants was recruited for the pilot test of the Moment for Parents prototype. Participants were recruited to the pilot test through personal networks and social media groups targeting pregnant and postpartum people who were interested in accessing mental health support. By recruiting these networks and online groups, we sought to reach a diverse range of users to ensure the prototype would be tested among people with different experiences in pregnancy and postpartum care.

#### Procedure

Data collection took place between November 2019 and June 2020. Upon enrollment, participants were provided with a no-cost, indefinite version of the Moment for Parents prototype, allowing participants to freely engage with the chatbot’s features without time constraints. After 1 month of using Moment for Parents, participants were invited to complete a survey that asked about their perceptions of the Web app (ie, prototype feedback survey).

#### Measures

##### Chatbot Engagement

Participants’ interactions with the Moment for Parents chatbot were tracked to assess usage patterns and calculate engagement metrics.

##### Prototype Feedback Survey

After 1 month of using Moment for Parents, participants completed a structured survey evaluating their experience with the chatbot. Participants received a text message inviting them to complete the survey, which included a link directing them to the questions in the Web app. Survey items assessed usability, perceived utility, relevance of content, and overall satisfaction. These responses allowed us to quantify participant satisfaction and identify trends in user experience.

### Data Analysis

Descriptive statistics were used to summarize participant characteristics, engagement patterns, and feedback survey responses. Means and SDs were calculated for continuous variables, while categorical variables were reported as frequencies and percentages. Engagement over time was analyzed by calculating the proportion of participants who remained active each month. Re-engagement patterns were assessed by identifying users who returned to the chatbot after at least 1 week of inactivity. Stata (version 15; StataCorp LLC) was used for the analyses.

## Results

### Phase 1 – Ethnographic Interviews

#### Participants

A total of 43 individual interviews were conducted. Demographic characteristics of participants included 56% (n=24) who were first-time pregnant mothers, 23% (n=10) who were mothers expecting their second or third child, and 21% (n=9) who were post partum with at least 1 child under the age of 1 year.

#### Qualitative Description Results

A summary of the key themes and subthemes derived from the qualitative description can be found in Table 1. The three main themes identified were (1) resources, (2) mental health journey, and (3) gaps in support. When asked what types of support users were looking for during the perinatal period, they reported seeking guidance from friends, family, peers, mental health professionals, and medical providers. Users also described the emotional trajectory they experienced, highlighting unique challenges present during the various phases of pregnancy and postpartum. Finally, users identified a variety of gaps in support that they felt could be addressed with a chatbot. These included a lack of reliable evidence-based psychoeducation on maternal mental health, social isolation from peers, and insufficient emotional support from medical professionals.

**Table 1.** Qualitative synthesis of findings from ethnographic interviews.

Theme		Description	Quotes
Resources^a^			
	Social support	Friends, family, and online communities eased anxiety through shared experiences and validation.	“I feel like I leaned a lot on my friends.” [P09^b^, postpartum, aged 10 mo] “It’s good to just hear opinions of other pregnant people instead of like professional opinions or thoughts.” [P22, pregnant, third trimester]
	Mental health tools	Apps and therapists provided emotional support, tracking, and coping strategies.	“I’ve been in touch with my therapist since late last year... It’s been really helpful just having an actual therapist to talk to and talk my anxieties out.” [P41, pregnant, second trimester]
	Clinical guidance	Health care providers and doulas were essential for reassurance, though often limited in time for deeper support.	"I listen to my doctor above all else. For my first birth, I didn’t want a C-Section, and they let me go three days and respected my wishes. I had such a positive experience with my health care team, which I know not everyone has, so I trust my doctor above all else.” [P07, pregnant, second trimester, aged 3 y]
Mental health journey^c^			
	Emotional trajectory	Anxiety, isolation, and the unpredictability of pregnancy and parenthood shaped mental health challenges. The pregnancy journey was marked by fluctuating emotions, anxiety, and adjustments in mental health.	“Mentally a very isolating time anyways – feel like unless you know someone close to you who is pregnant around the same time, even in normal world standards, it can be very isolating.” [P16, pregnant, third trimester] “I’m not as happy as I was in the second trimester, I feel always tired. A little bit more emotionally trying, especially since I can’t hang out with my friends.” [P02, pregnant, third trimester]
	Early pregnancy stress	Intense physical symptoms and fear of complications caused heightened anxiety in the first trimester.	“Trying to think that you know I don’t want the baby to feel this. I want the baby to know that she’s loved and don’t want her to feel affected by the situation.” [P40, postpartum, aged 15 mo]
	Late pregnancy anxiety	Worries about labor and delivery escalated in the third trimester, along with physical discomfort.	“You’re supposed to be happy about this, it shouldn’t be terrible, not a walk in the park, but unknowns surrounding childbirth have been the biggest worry in back of my mind” [P30, pregnant, third trimester]
	Postpartum adjustment	Burnout, hormonal changes, and lack of support created emotional strain during the postpartum period.	“Honestly, I was burned out. I was giving and giving, and it felt like I had nothing left for myself.” [P15, postpartum, aged 10 mo] “I think the hardest part for me postpartum was not taking care of the baby but how do I take care of myself.” [P40, postpartum, aged 15 mo]
Gaps in support^d^			
	Maternal mental health	Overemphasis on baby care left mothers feeling unsupported in managing their own emotional well-being.	“... would have been nice to have more information on raising kids developmentally along with doing things that help me cope with boiling of stress.” [P07, pregnant, second trimester, aged 3 y] “All the apps that I have are pretty much focused on the baby. Besides the workout one, I would say there’s not an app that’s more focused on the mother.” [P11, postpartum, aged 5 mo]
	Community isolation	Young or first-time moms felt isolated and lacked connections to peers for emotional support.	“I’ve never been around people that are pregnant and haven’t spent much time around little babies so I feel ignorant to most things regarding pregnancy, giving birth and having a child. Starting from complete scratch…” [P01, pregnant, second trimester]
	Limited time and support from clinicians	Frustration with short, impersonal doctor visits that prioritized medical information over emotional support	“[Appointments are so short] that I didn’t want to bother my doctor all the time with little things I was feelings.” [P16, pregnant, third trimester] “Yeah, the first few months were hard and I felt like that was when I needed the most like reassurance that like ‘This is temporary.’” [P26, postpartum, aged 1 y, aged 4 y]
	Lack of understandable and credible sources	Frequently encountered misinformation online and desired reliable, easy-to-understand sources.	"Message boards mostly don’t work for me -- joined some of the What to Expect forums, and 75% of the time they lead you down such a rabbit hole, people have such different opinions and its not helpful and maybe scares you more. 25% of the time I like them because knowing a bunch of people who are going through the same things is helpful.” [P35, pregnant, second trimester, aged 3 y]

a Integration into prototype: Moment for Parents was designed to offer validation, reassurance, and coping strategies for mothers. Developed by emotional well-being experts—including a doula, perinatal therapist, and yoga and meditation instructor—it incorporated real-life examples from mothers to highlight shared experiences.

b P: participant.

c Integration into prototype: Moment for Parents included acceptance and commitment therapy exercises to boost self-acceptance and psychological flexibility, helping moms process emotions such as isolation and anxiety. It also used cognitive behavioral therapy techniques to address worries about physical symptoms and mindfulness practices for coping with discomfort. During each check-in, the chatbot encouraged users to create a self-care plan and suggested activities lasting 5, 15, 30, and 60 minutes.

d Integration into prototype: Moment for Parents focused entirely on mothers’ emotional and mental well-being, reinforcing the importance of self-care in supporting their baby. The chatbot guided moms in building a support system through exercises on accepting and asking for help, challenging the “do it all” myth, and promoting a mindset of community care.

#### Prototype Development

The results from the analysis of the ethnographic interviews directly influenced the content in the first Moment for Parents prototype. First, the “resources” theme resulted in exercises aiming to provide validation, reassurance, and coping strategies for users. Given specific user needs around social support, Moment for Parents also included real examples from parents with mental health struggles to illustrate shared experiences. Second, results from the “mental health journey” theme inspired acceptance and commitment therapy based exercises around self-acceptance and psychological flexibility. The goal for these exercises was to help users process their negative emotions, including anxiety and isolation, that users reported experiencing throughout the perinatal period. The “mental health journey” theme also led to the development of exercises tailored to the different phases of the perinatal period. For example, cognitive behavioral therapy exercises to increase coping with worries about physical symptoms in early pregnancy, and exercises focused on increasing self-care exercises during the postpartum period. Finally, the “gaps in support” theme led to the inclusion of exercises designed to help users increase help-seeking and build their support systems. The multidisciplinary team of experts vetted and fleshed out the content based on the user interview themes. Other aspects of the prototype, such as the structure (eg, frequency of messaging), were also informed by insights from the team of experts.

The first prototype consisted of a rules-based chatbot called Moment for Parents embedded within a Web app. Upon logging in, participants completed a mood check-in. Based on their mood at the check-in, the chatbot offered an exercise tailored to their current mood. The Moment for Parents prototype had a library of 35 exercises that the chatbot would select based on the users’ reported mood. A second version of the prototype was adopted after 4 weeks based on participant feedback. In this version, participants were offered the option to select their own journey through the app. Options included the following: (1) an exercise based on mood, (2) an exercise on a predetermined topic (ie, “topic of the day”), and (3) a self-care check-in. The following day, the chatbot followed up with participants to encourage accountability and engagement in the exercises provided by the chatbot.

### Phase 2 – Pilot Test

#### Participant Characteristics

A total of 108 participants, 29 pregnant and 79 post partum, participated in the pilot test of the Moment for Parents prototype. Among pregnant participants, 69% (n=20) were experiencing their first pregnancy, while 31% (n=9) were pregnant for their second time or more. Additionally, 17% (n=5) of pregnant participants were in their first trimester, 31% (n=9) in their second trimester, and 52% (n=15) in their third trimester. For postpartum participants, 82% (n=65) had 1 child, and 18% (n=14) had multiple children. Additionally, 25% (n=20) participants were 0‐13 weeks post partum, 25% (n=20) participants were 14‐26 weeks postpartum, 17% (n=13) participants were 27‐40 weeks postpartum, and 33% (n=26) participants were 41‐52 weeks postpartum.

#### Overall Engagement

Participants completed a total of 1036 check-ins and exercises between November 2019 and June 2020, with an average of 9.6 (SD 14.5) check-ins per participant and a mean of 4.83 weeks (SD 5.17) of engagement. A total of 31.9% (n=331) of check-ins occurred during the first 2 weeks, and 46.1% (n=478) occurred within the first month. Over the second month, check-ins dropped to 17.5% (n=181), followed by 9.6% (n=99) in the third month. During the fourth and fifth months, 6.5% (n=67) of check-ins occurred each month, followed by a slight rise to 6.8% (n=68) in the sixth month. Check-ins then decreased to 2.9% (n=30) in the seventh month and 1.0% (n=10) in the eighth month.

Table 2 presents the number of participants who engaged with the Web app at least once within each given month for all participants and separately for pregnant and postpartum groups. At 1 month, nearly all participants (98.1%) remained active, with higher retention among postpartum users (100%) compared to pregnant users (93.1%). Retention remained high at 2 months, with almost half (47.2%) of users still engaged. While engagement declined over time, postpartum users showed sustained interest, with 15.2% active at 7 months and 7.6% at 8 months.

**Table 2.** Moment for parents monthly active users over the 8-month evaluation (n=108).^a^

	All (N=108), n (%)	Pregnant (n=29), n (%)	Postpartum (n=79), n (%)
1 month	106 (98.1)	27 (93.1)	79 (100)
2 months	51 (47.2)	9 (31.0)	42 (53.2)
3 months	22 (20.4)	3 (10.3)	19 (24.1)
4 months	17 (15.7)	3 (10.3)	14 (17.7)
5 months	22 (20.4)	4 (13.8)	18 (22.8)
6 months	14 (13)	1 (3.4)	13 (16.5)
7 months	12 (11.1)	—^b^	12 (15.2)
8 months	6 (5.6)	—	6 (7.6)

a Most users (n=90, 83.3%) were active during their first week of use; 47.2% (51/108; 9/29, 31% pregnant, 42/79, 53.2% postpartum) used the app during their second week.

b Not available.

#### Re-Engagement Patterns

A total of 25 (5 pregnant and 20 postpartum) participants used the app for a median of 1 week (mean 1.8, SD 3.33 wk) before stopping and never returning. In contrast, 69 (14 pregnant and 55 postpartum) participants eventually re-engaged with the app after at least one week of inactivity. Among these participants, 44.9% (31/69) returned only once (8/14, 57% of pregnant participants; 23/55, 42% of postpartum participants). Re-engagement was operationalized as the number of times participants returned to the app after stopping for at least 1 week of inactivity. Of participants who re-engaged, a large proportion (28/69, 41%) did so 3 or more times after at least 1 week of inactivity. Postpartum participants re-engaged more often and more quickly than pregnant participants. Most re-engagements took place between 1 and 2 weeks after initial re-engagement, but a significant percentage of re-engagements occurred after 4 weeks (58/402, 14%; Table 3).

**Table 3.** Re-engagement patterns among those who re-engaged with moment for parents after stopping (n=69 participants; n=402 check-ins).

		All	Pregnant	Postpartum
Time to re-engagement (week), mean (SD)^a^		2.8 (2.6)	3.2 (3.2)	2.6 (2.5)
Number of re-engagements, n (%)^a^				
	1 re-engagement	31 (44.9)	8 (57.1)	23 (41.8)
	2 re-engagements	10 (14.5)	2 (14.3)	8 (14.5)
	3+ re-engagements	28 (40.6)	4 (28.6)	24 (43.6)
Number of check-ins after re-engagement, n (%)^b^				
	After 1 week	160 (39.8)	28 (38.9)	132 (40)
	After 2 weeks	106 (26.4)	19 (26.4)	87 (26.4)
	After 3 weeks	78 (19.4)	15 (20.8)	63 (19.1)
	After 4+ weeks	58 (14.4)	10 (13.9)	48 (14.5)

a All (n=69), pregnant (n=14), and postpartum (n=55).

b All (n=402), pregnant (n=72), and postpartum (n=330).

#### Prototype Feedback

Feedback from users (n=30) who completed the 1-month survey is in Table 4. Most users signed up for Moment for Parents because a friend recommended it (n=15, 55%). Other top reasons for signing up included an interest in becoming more resilient (n=14, 47%), wanting support for a self-care routine (n=13, 43%), and looking for advice to take care of themselves (n=12, 40%). The vast majority (n=28, 93%) felt that Moment for Parents was created for people such as them. Most users thought that Moment for Parents was a good fit for them because it kept them accountable for self-care (n=21, 70%), it reminded them to reflect (n=19, 63%), and it helped them take a mental health break (n=17, 58%). A large proportion of users also felt that Moment for Parents validated what they were going through (n=13, 43%), gave helpful advice (n=13, 43%), and helped them navigate the ups and downs of motherhood (n=12, 40%). While users generally had a positive experience with Moment for Parents, some users reported several “pet peeves” or negative experiences. Half of the users felt that the check-in topics were repetitive (n=15), and 37% (n=11) of users reported a pet peeve when the check-ins had a glitch. A third of users felt that the check-ins did not have the response options they wanted (n=10). Less frequently reported pet peeves included the daily text reminders (n=8, 27%), the lack of access via a mobile app (n=6, 20%), and feeling as if the check-in topic was not relevant (n=6, 20%). The vast majority of users spent less than 5 minutes completing their check-ins (n=28, 93%), and most (n=24, 80%) felt that the check-in length was “just right.”

**Table 4.** Feedback from users who completed the 1-month survey (n=30).

Feedback		Value, n (%)
Why did you sign up for Moment for Parents?		
	Friend recommended	15 (55.3)
	Interested in becoming more resilient	14 (46.7)
	Interested in trying a new product for moms	9 (30)
	Looking for advice to take care of baby	4 (13.3)
	Looking for advice to take care of myself	12 (40)
	Help coping with emotions	10 (33.3)
	Support for self-care routine	13 (43.3)
	Other	1 (3.3)
Was Moment for Parents created for moms like you?		
	Yes	28 (99.3)
Why is Moment for Parents a good fit for you?		
	Feels like a friend	11 (36.7)
	Gives me confidence as a mom	5 (16.7)
	Gives me helpful advice	13 (43.3)
	Helps me become resilient	7 (23.3)
	Helps me navigate ups and downs of motherhood	12 (40)
	Helps me take a mental health break	17 (56.7)
	I know it’s there in case I need it	1 (3.3)
	It keeps me accountable for self-care	21 (70)
	It offers helpful mindfulness exercises	9 (30)
	It reminds me to reflect	19 (63.3)
	It surprises me with new perspectives	6 (20)
	It understands what I’m going through	7 (23.3)
	It validates what I’m going through	13 (43.3)
What are your pet peeves about Moment for Parents?		
	Can’t access via an app	6 (20)
	Daily text reminders	8 (26.7)
	None–it’s great as-is	3 (10)
	Not enough support for sleep	4 (13.3)
	Tone is too empathetic	1 (3.3)
	Something else	5 (16.7)
	Too much emphasis on self-care	3 (10)
	When check-in has a glitch	11 (36.7)
	When check-in questions don’t have the response option I want	10 (33.3)
	When texts come too quickly	3 (10)
	When texts come too slowly	1 (3.3)
	When check-in topic doesn’t feel relevant to me	6 (20)
	When check-in topics are repetitive	15 (50)
	When Moment for Parents doesn’t respond to me appropriately	5 (16.7)
Time to complete check-in		
	Less than 1 min	3 (10)
	1‐3 min	20 (66.7)
	3‐5 min	5 (16.7)
	More than 5 min	2 (6.7)
Check-in length		
	Just right	24 (80)
	Too long	5 (16.7)
	Too short	1 (3.3)

## Discussion

### Summary of Findings

The purpose of this study was to (1) design and develop a chatbot tailored specifically to educate and support pregnant and postpartum people on emotional well-being and mental health and (2) evaluate the usability of Moment for Parents in a pilot test by examining usage patterns, engagement levels, and user experience. In the design phase, we found that user needs included social support, coping strategies, more attention to mental health and self-care, and reliable medical and mental health information. Users also emphasized a need for support that is tailored to changes over the perinatal period. The end result of phase 1 was an initial prototype of the Moment for Parents chatbot that was used in the phase 2 pilot test. The results from the pilot test showed that participants engaged with the app early on and that sustained engagement decreased over time. However, many participants re-engaged with the chatbot after inactivity, suggesting that users may have turned to Moment for Parents when they needed support. The results from the feedback survey showed that participants were primarily interested in using Moment for Parents to build resilience, support their self-care routines, and seek emotional support during pregnancy and postpartum.

Themes from the qualitative analysis highlighted the key needs and preferences of perinatal users. Participants consistently emphasized the importance of evidence-based strategies to support their mental health, particularly as a complement to in-person care, where provider time for emotional support may be limited. Additionally, users described the fast-paced and unpredictable nature of the perinatal period, reinforcing the need for flexible and adaptive digital interventions that align with their evolving emotional and psychological needs. These findings underscore the importance of delivering personalized, flexible, and easily accessible digital support that aligns with the evolving needs of perinatal individuals. Such alignment may also play a critical role in sustaining user engagement over time.

In the pilot trial, user engagement was greatest in the first 2 months, then decreased over the next 6 months, both in terms of check-ins with the app and monthly active users. Importantly, 47% of users were still active after 2 months—this level of usage is much greater than metrics described in other publicly available mental health applications where users drop to only 4% of the initial sample after 2 weeks [39]. There are several possible explanations for this finding. First, Moment for Parents was developed using HCD, which is thought to enhance engagement with digital mental health interventions [40]. Feedback showed that most participants felt that the information and exercises in Moment for Parents were highly personalized and relevant, which may have led to more sustained engagement. Another possible explanation is that, given the time-consuming demands faced by perinatal individuals such as attending medical appointments, managing physical symptoms, breastfeeding, and childcare, engaging with Moment for Parents required relatively little time and energy from users, as most users reported spending less than 5 minutes with Moment for Parents daily. This is in contrast to other apps and chatbots that are more time and resource intensive [41,42].

While most participants disengaged after 2 months, many re-engaged with Moment for Parents at least once during the pilot. Among those who returned, a large proportion did so 3 or more times, suggesting they found the app useful or relevant to their needs. Re-engagement was highest in the first week (40%), but a notable portion (14%) continued to return after 4 weeks, indicating that users may have turned to the app specifically when they needed support or information. A recent review of 16 trials of digital mental health interventions for perinatal populations found that disengagement varied widely across trials (13%‐90% retention rates) and highlights the challenge of engaging perinatal users [43]. Moment for Parents was designed to deliver targeted support based on mood check-ins rather than continuous interaction, highlighting the importance of minimal contact and flexible mental health tools. Future research should prioritize understanding the timing and reasons behind user re-engagement, rather than solely aiming to increase engagement frequency, to enhance the mental health chatbots’ ability to deliver timely, relevant support tailored to users’ needs. This could include exploring the specific triggers for re-engagement, the impact of intermittent use on mental health outcomes, and how to refine content delivery to maximize effectiveness when users do return.

Interestingly, both engagement and re-engagement were greater among postpartum participants compared to pregnant participants. Postpartum users may have been experiencing greater mental health challenges than their pregnant counterparts in this sample and, therefore, turned to the app more frequently for exercises to improve their mood. Relatedly, postpartum users may have sought more information from the app as they learned to care for their infants, managed their postpartum mental health, and adjusted to new parenthood. Additionally, the postpartum period is marked by significant hormonal changes, sleep deprivation, and increased caregiving demands, all of which contribute to low mood [44-46]. Given these challenges, postpartum individuals may be more open to digital mental health support as a convenient and accessible resource for coping strategies, reassurance, and parenting guidance [47]. In contrast, pregnant users may have relied more on in-person medical support or other existing resources, leading to lower engagement with the app. Alternatively, some users indicated in informal feedback that the content presented in Moment for Parents was geared toward postpartum more than pregnant user needs. Overall, the limited number of pregnant individuals in our sample prevents definitive conclusions, highlighting the need for further research on tailoring interventions to pregnant user needs.

The findings from the 1-month feedback survey reinforced the value of Moment for Parents in supporting perinatal mental health. The users reported that the tool provided helpful advice, supported self-care accountability, and facilitated emotional reflection, highlighting its role in bridging gaps in traditional care [48,49]. Many found the content relevant and beneficial, though some noted frustration with repetitive or irrelevant check-ins, emphasizing the need for customizable, phase-specific content. These findings underscore the potential of personalized digital interventions to enhance accessibility and engagement in perinatal mental health support.

### Limitations

This study was not without limitations. First, the samples used in both phases of this study were relatively small and prone to bias because of the snowball sampling method used. Certain groups or participant characteristics may have been overrepresented, leading to skewed results. Furthermore, only 25% of the initial sample completed the 1-month survey, leading to potentially biased survey results if, for example, only users who enjoyed the app completed the survey. Relatedly, another limitation was that no demographic data was collected about study participants in either phase, limiting our ability to speak to the generalizability or biases in the samples studied. Second, the sample primarily consisted of postpartum users, limiting our ability to make inferences about the feasibility and utility of Moment for Parents across the perinatal period. Future work should focus on recruiting a larger sample of pregnant users at various stages of pregnancy, as well as postpartum users at various postpartum stages (eg, early postpartum vs late postpartum). Third, the way the feedback questions were asked may have led participants to provide overly positive feedback (eg, “why was Moment for Parents a good fit” vs “to what extent do you agree that Moment for Parents was a good fit?”). Future evaluations of Moment for Parents should include a more balanced survey to capture a variety of perspectives. Fourth, systematic data were not collected around the nature and timing of the iterative updates, which limits our ability to understand how these updates impacted engagement or satisfaction with Moment for Parents. Finally, data were not collected around user enactment of the Moment for Parents exercises outside of the check-ins. Future research should focus on understanding how users integrate the strategies they learn from Moment for Parents into their daily lives.

### Conclusions

This study provides early evidence that Moment for Parents is a practical and scalable tool for perinatal mental health support. HCD informed the chatbot’s development, ensuring it aligned with the needs of perinatal users before being tested in a pilot trial. Findings showed that participants engaged with the chatbot far longer than is typical for digital interventions and often returned for support even weeks after initially stopping use—highlighting its usability and value. However, more research is needed to fully understand the chatbot’s efficacy in mitigating adverse perinatal mental health outcomes. These results contribute to the growing body of research on digital mental health interventions in the perinatal period, reinforcing the potential of chatbots to deliver accessible, on-demand support.

## Abbreviations

BRANY: Biomedical Research Alliance of New York
HCD: human-centered design
